# Transport of Moving Duck Flocks in Indonesia and Vietnam: Management Practices That Potentially Impact Avian Pathogen Dissemination

**DOI:** 10.3389/fvets.2021.673624

**Published:** 2021-07-09

**Authors:** Shan Wen Stacy Khaw, Le Tri Vu, Didik Yulianto, Joanne Meers, Joerg Henning

**Affiliations:** ^1^School of Veterinary Science, The University of Queensland, Gatton, QLD, Australia; ^2^Regional Animal Health Centre VI, Ho Chi Minh City, Vietnam; ^3^Disease Investigation Centre, Wates, Indonesia

**Keywords:** avian influenza, transport, moving ducks, biosecurity, virus transmission, Indonesia, Vietnam

## Abstract

Highly pathogenic avian influenza (HPAI) virus is endemic in Indonesia and Vietnam, where “moving” duck production is commonly practiced. Questionnaire surveys were conducted with transporters of “moving” duck flocks in Indonesia (*N* = 55) and Vietnam (*N* = 43). The main purpose of transportation was to transport duck flocks between rice paddies used for scavenging. Trucks were commonly utilized for transport in both countries (Indonesia: 98.2%, 54/55; Vietnam: 37.2%, 16/43), while boats were only used in Vietnam (62.8%, 27/43). Transporters in Vietnam moved larger flocks and traveled over longer distances. Deaths of ducks due to diseases were reported in both countries (Indonesia: 16.4%, 9/55; Vietnam: 4.7%, 2/43; *p* = 0.11). Throwing away of carcasses was the primary method of disposal of dead birds in Indonesia (60.0%, 33/55), but was not practiced in Vietnam (*p* < 0.001), while more transporters in Vietnam (34.9%, 15/43) buried carcasses compared to Indonesia (6.8%, 4/55; *p* = 0.001). Consumption of carcasses (20.9%, 9/43), sale of dead ducks (14.0%, 6/43) and processing of ducks for fish feed (9.3%, 4/43) was conducted in Vietnam, but not in Indonesia. Vehicles were predominantly cleaned in rivers and stored outside in Vietnam, while cleaning and storage was usually conducted in houses/garages in Indonesia. In conclusion, we identified management practices that potentially impact transmission of avian pathogens, such as HPAI virus. In Indonesia, unsafe management practices were related to multipurpose usage of transport vehicles and disposal of birds in the environment, while in Vietnam, they were related to the mixing of birds during transport, the processing of dead carcasses and the storage and cleaning of transport vehicles.

## Introduction

Highly Pathogenic Avian Influenza (HPAI) H5N1 virus is endemic in Vietnam and Indonesia and has caused substantial human and poultry losses (1, 2). From 2003 to September 2018, 454 human fatalities were reported out of 860 HPAI human cases worldwide, representing a case-fatality rate of 52.8% (3). At 84.0% (168/200), Indonesia has the highest human case fatality rate globally (3). Vietnam has experienced 64 deaths out of 127 human cases, representing a case fatality rate of 50.4% (3).

From 2004 to 2019, 22.5 and 20.0% of the global HPAI outbreaks in poultry occurred in Indonesia and Vietnam, respectively (4). Since 2003, more than 150 million domestic birds died or were culled as a result of H5N1 outbreaks in Indonesia (5, 6), while in Vietnam more than 52 million poultry losses occurred since 2003, with 86.5% of the domestic poultry population being culled in 2003-04 alone at an estimated cost of about US$205 million (7).

Duck farming is an important sector of the poultry industry in Indonesia and Vietnam. Duck management is classified into “stationary” and “moving” duck production, with stationary flocks allowed to graze around the village vicinity and secured at night near village houses, while moving flocks are moved between areas of recent rice harvests and kept in confinement overnight close to the daytime scavenging locations (8–10). Moving duck flocks are suspected to contribute toward the maintenance and circulation of HPAI viruses (11–14) and research had highlighted that road characteristics (e.g., road density; road length), human and poultry densities and long distance movement might facilitate the spread of HPAI viruses (15–19). Meyer et al. (20) described the actors involved in duck production, providing an overarching description of the poultry value chain system of Vietnam, while Henning et al. (9) described the structure of the moving duck flock network in Indonesia. However, specific transport practices that influence the dissemination of H5N1 virus have yet to be identified.

The objectives of this study were to (1) identify management factors during transport of moving duck flocks in Indonesia and Vietnam that could potentially be associated with an increased risk of avian pathogen dissemination (e.g., HPAI virus), and to (2) compare and contrast differences in movement, management and biosecurity practices implemented by duck flock transporters in Indonesia and Vietnam.

## Materials and Methods

### Study Design

Previous research conducted with moving duck flock owners in Indonesia and Vietnam in 2008 and 2009 described the HPAI H5N1 infection status of ducks and the movements elected by owners for their duck flocks (8, 9). During this research, information about the transport provider used by moving duck farmers was collected. These data comprised the initial dataset of transporters to be contacted. As no register of transporters existed in either country, we used snowball sampling by asking identified transport providers about contact details of additional potential transporters working in the same region. Transporters were identified in six districts of Central Java (Pemalang, Batang, Klaten, Purworejo, Brebes, and Kendal) in Indonesia and in four provinces of the Mekong Delta in Vietnam (Ben Tre, Dong Thap, Tien Gian, and Vinh Long). The aim was to interview about 10 transporters per district or province.

Data collection was conducted in Indonesia by veterinarians from the Disease Investigation Centre (DIC) in Wates, Yogjakarta and in Vietnam by veterinarians from The Regional Animal Health Centre VI, Ho Chi Minh City using an interview process using local languages. All interviewers were trained in data collection.

The study design for this research was reviewed and approved in Indonesia by the Disease Investigation Centre (DIC) in Wates, Yogjakarta; and in Vietnam by the Regional Animal Health Centre VI, Ho Chi Minh City. Data collection for this study was conducted in accordance with the accepted survey guidelines for surveillance activities of both organizations.

### Questionnaire

Questionnaires were developed in English and later translated into the national languages (Bahasa, Vietnamese) in order to capture potential associated risk factors associated with spread of avian pathogens such as HPAI virus during the transport of duck flocks: type of transport utilized; number of flocks (and ducks) transported per time period; age of ducks transported; number of duck flocks combined in a transport load; other poultry species transported; number of farms visited to obtain one transport load; cleaning and disinfection before and after transport; location where the transport vehicles were stored, cleaned and disinfected; distance and duration of transport; management of ducks before departure, during transport and after arrival in scavenging area; contact of transported ducks with other poultry and other animals; frequency of transporting ducks; raising of ducks at home by people loading and transporting poultry; transport of items (e.g., chickens, other animals, feed, and eggs) together with ducks; experiences of sickness or deaths of ducks during transport; disposal of dead ducks; and occurrence of health problem in people loading and transporting ducks. Thus, the questionnaires included a mixture of closed and open-ended questions. Copies of the questionnaires are provided in the Supplementary Material (Data Sheets 1, 2).

Pilot testing of the questionnaires was performed with two transporters in both countries before conducting the main survey to identify any problems, misunderstandings or to discover additional risk factors of interest that should be surveyed. The questionnaires were updated accordingly. The total number of questions in the questionnaire was 39, with identical questions being used in Indonesia and Vietnam.

Data analysis was conducted in SPSS (IBM Corp, Release 2019, IBM SPSS Statistics for Windows, Version 26.0) and STATA (StataCorp, College Station, TX, 2019, Stata Statistical Software: Release 16). Descriptive analysis included the calculation of frequencies, means, medians and range values. The command –*tabplot*- in Stata was used to visualize the frequency of responses provided on a 4-point Likert scale. The total number of survey responses for each response category were compared between Indonesia and Vietnam using the Fisher's exact test. To facilitate the utilization of the Fisher's Exact Test for data analysis, Likert scale groups “very important” and “important” were combined into a category “importantly” and Likert scale groups “not important” and “not conducted” were combined into a category “not importantly.” Similarly, Likert scale groups “common” and “sometimes” were combined into a category “commonly” and Likert scale groups “seldom” and “not conducted” were combined to a category “infrequently.” The non-parametric Mann–Whitney *U*-test was used to compare ordinal and not normally distributed continuous variables between Indonesia and Vietnam.

## Results

A total of 114 transporters of moving duck flocks were interviewed, with 16 transporters being excluded from the analysis as they provided incomplete information in the questionnaire. Thus, 98 transporters provided completed responses to all questions and were analyzed in detail, consisting of 55 transporters from Indonesia and 43 from Vietnam (Supplementary Table 1 - Data Sheet 3).

### Importance of Transport Activities for Income Generation

**Figure 1:**
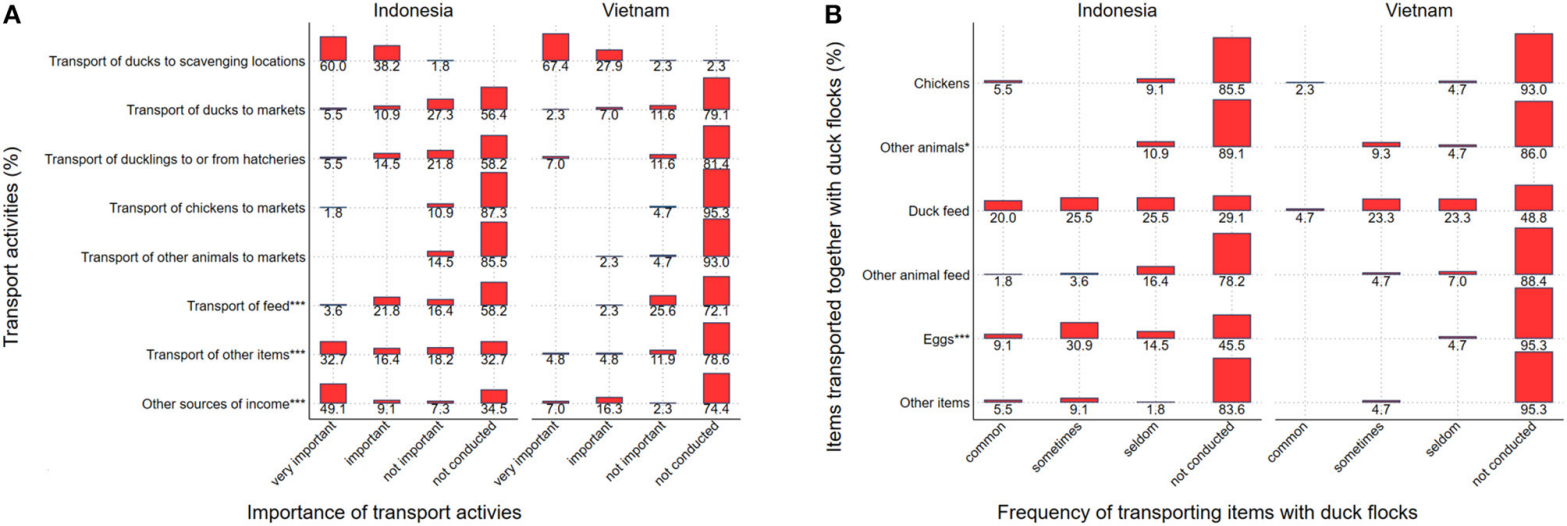
**(A)** Importance of transport activities for transporters of moving duck flocks in Indonesia (*N* = 55) and Vietnam (*N* = 43), with percent of respondents by importance category. **(B)** Items transported together with ducks to scavenging locations for Indonesia (*N* = 55) and Vietnam (*N* = 43), with percent of respondents by frequency category. *P*-values for comparisons between countries for each category are presented as follows: * for *P* ≤ 0.05, *** for *P* ≤ 0.001.

As expected, the transport of ducks to scavenging locations was the main activity for transporters (Figure 1A) in both countries (Indonesia: 98.2%, 54/55, Vietnam: 95.3%, 41/43; *p* = 0.58). Although not statistically significant, transporting of ducks to markets was less common in Indonesia (Indonesia: 9.3%, 4/43; Vietnam: 16.4%, 9/55; *p* = 0.38), while transport of ducklings to and from hatcheries was more common in Indonesia. Items transported together with ducks include chickens, other birds, feed, and eggs (Figure 1B). About 45.5% of transporters from Indonesia indicated that they “commonly” transport duck feed together with ducks compared to only 28.0% of transporters from Vietnam (*p* = 0.09). Additionally, 40.0% of transporters from Indonesia indicated that they “commonly” transport eggs together with ducks, but this was either seldom or not practiced in Vietnam (*p* < 0.001) (Figure 1B).

In general, transporters from Indonesia also used their transport vehicles more frequently to transport other items for income generation. This included the transport of feed (Indonesia: 25.5%, 14/55; Vietnam: 2.3%, 1/43; *p* = 0.0014), and transport of non-animal related items (Indonesia: 49.1%, 27/55; Vietnam: 9.5%, 4/42; *p* < 0.001), with the most common items transported being building materials (Figure 1A). More Indonesian transporters relied on additional income sources not related to transport (Indonesia: 58.2% 32/55; Vietnam: 23.3%, 10/43; *p* < 0.001), with farming being the most common activity in both countries (Figure 1A).

### Transport Types Used, Volume of Transport and Distance Traveled

Trucks, boats and motorbikes were used for transportation (Supplementary Figure 1 - Data Sheet 3). Trucks were the most common transport type in Indonesia (Indonesia: 98.2%, 54/55; Vietnam: 37.2%, 16/43; *p* < 0.001), while boats were the most common mode of transport in Vietnam (62.8%, 27/43), but were not utilized in Indonesia (*p* < 0.001). Motorbikes were used by one transporter in each country. Almost all transporters (99.0%) utilized only one type of transport.

Of the 70 transporters, who provided data on truck designs, 95.7% (67/70) of trucks were not covered or closed. Trucks had between 1 and 4 levels, with the majority of them (58.6%, 41/70) having three levels. Boats were generally open (82.1%, 23/28) and had between 1 and 3 levels, with 85.7% (24/28) of them having three levels. Neither of the two motorbikes was covered.

Respondents from Vietnam were more likely to store their transport vehicles outside (60.5%, 26/43) compared to Indonesia (16.4%, 9/55; *p* < 0.001). Vietnamese transporters indicated more frequent exposure of their vehicles to wild birds compared to Indonesian transporters [23.3% (10/43) vs. 5.5% (3/55); *p* = 0.015].

The volume of transport and distance traveled in Indonesia and Vietnam are shown in Table 1. In Vietnam, transporters using trucks transported more duck flocks per year, over larger distances per year and with a larger number of ducks per flock compared to Indonesia. Individual journeys were ~25% longer in Vietnam compared to Indonesia, although this was not significant (*p* = 0.13).

**Table 1:** Volume of transport and distance traveled by transporters of moving duck flocks in Indonesia.

	**Transport by truck**			**Transport by boat**
	**Indonesia**	**Vietnam**	***p*-value**	**Vietnam**
Number of scavenging duck flocks transported per year	44 (48; 2–270)	123 (110; 50–220)	<0.001	113 (120; 40–250)
	(*N* = 45)	(*N* = 14)		(*N* = 28)
Number of ducks transported in a single load	431 (450; 100–800)	4,792 (750; 300–25,000)	<0.001	18,527 (12,000; 800–80,000)
	(*N* = 51)	(*N* = 13)		(*N* = 26)
Distance traveled per year (in km) to scavenging locations	1,831 (800; 80–20,000)	9,483 (10,000; 900–35,000)	<0.001	10,450 (6,000; 50–55,000)
	(*N* = 37)	(*N* = 15)		(*N* = 26)
Distance traveled per journey (in km) to scavenging locations	68 (60; 10–200)	94 (80; 25–200)	0.13	97 (80; 10–300)
	(*N* = 45)	(*N* = 16)		(*N* = 26)

*Mean (median; minimum - maximum) is shown with number of respondents in brackets. P-value refers to the comparison between Indonesia and Vietnam*.

### Transport of Moving Duck Flocks to and From Scavenging Locations

The locations from where ducks were collected from and transported to by transporters are shown in Supplementary Figure 2 (Data Sheet 3). As expected, the majority of transporters from both countries collected ducks from rice paddies as these represent the main scavenging locations. However, 78.2% (43/55) of transporters in Indonesia collected ducks and 63.0% (34/54) delivered ducks to farms, compared to only 23.3% (10/43) and 20.9% (9/43) from Vietnam (*p* < 0.001 and *p* < 0.001, respectively). About 34.5% (19/55) of transporters in Indonesia collected and 53.7% (29/54) delivered moving ducks to village areas compared to 58.1% (25/43) and 65.1% (28/43) in Vietnam (*p* = 0.025 and *p* = 0.30, respectively), highlighting that within-village scavenging areas are more common in Vietnam.

Collection and delivery of ducks to markets was uncommon for both Indonesia and Vietnam, with about 11% and < 5% of transporters in Indonesia and Vietnam, respectively, “commonly” conducting this practice.

Characteristics of return journeys are outlined in Supplementary Figure 3 (Data Sheet 3). Although usually transporters from both countries returned “empty” after delivering ducks, 34.9% (15/43) of transporters in Vietnam and 20.4% (11/54) of transporters from Indonesia (*p* = 0.17) did “commonly” return with other ducks.

A higher proportion of transporters in Vietnam compared to Indonesia provided care to birds during transport. In Indonesia, water and feed was provided to ducks by 13.0% (7/54) and 7.4% (4/54) of transporters, respectively, compared to 88.4% (38/43) and 65.1% (28/43) of transporters, respectively, in Vietnam. Spraying of birds with water was conducted by 5.6% (4/54) of transporters in Indonesia and 32.6% (14/43) of transporters in Vietnam, while rest stops for ducks were provided by 11.1% (6/54) of transporters in Indonesia and 18.6% (28/43) of transporters in Vietnam.

### Duck Deaths and Disposal of Carcasses

Out of 41 transporters providing information on the number of duck deaths per truck load in Indonesia, the mean number (median, range) of duck deaths per load was 2.4 (2, 0.5–10), while for Vietnam, out of 16 transporters providing information, the mean number (median, range) of duck deaths per truck load was 3.4 (3, 1–10). For 26 transporters with boats from Vietnam who provided data, the mean number (median, range) of duck deaths per load was 2.6 (2, 1–5).

**Figure 2:**
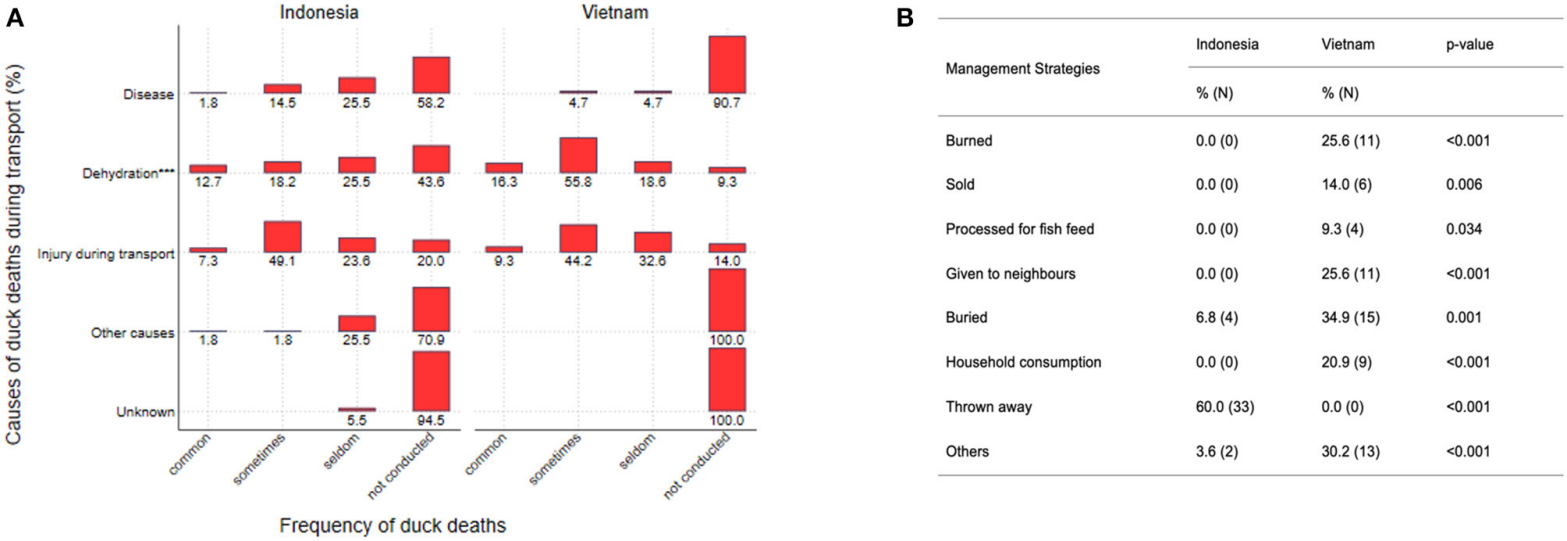
**(A)** Causes of death ducks during transport in Indonesia (*N* = 55) and Vietnam (*N* = 43) with percent of respondents by frequency category. *P*-values for comparisons between countries for each category are presented as follows: *** for *P* ≤ 0.001. **(B)** Disposal of ducks that died during transport in Indonesia and Vietnam.

Transporters reported the causes of death as “disease,” “dehydration,” “injury,” “other,” and “unknown.” Deaths of ducks during transport due to diseases were observed by transporters in both countries at similar frequencies (Figure 2A, *p* = 0.11). Injuries occurred in similar frequencies during transport in Indonesia and Vietnam (*p* = 0.84). In contrast, transporters from Vietnam more frequently experienced death of ducks due to dehydration (Vietnam: 72.1%, 31/43; Indonesia: 30.9%, 17/55; *p* < 0.001).

There were considerable differences in how transporters disposed of ducks that died during transport (Figure 2B). Throwing away carcasses in the environment was most common in Indonesia (60.0%, 33/55), but not practiced in Vietnam (*p* < 0.001). Sale of dead ducks (*p* = 0.006), processing of ducks for fish feed (*p* = 0.034), giving ducks to neighbors (*p* < 0.001), and household consumption of ducks (*p* < 0.001) were all practiced in Vietnam, but not in Indonesia (Figure 2B).

In Indonesia, two transporters specified as “other” management practice that duck carcasses were returned to duck owners (3.6%, 2/55). For Vietnam, “other” management practices also included return of carcasses to duck owners (20.9%, 9/43), while 9.3% (4/43) of transporters provided carcasses to para-veterinarians.

### Cleaning and Disinfection of Transport Vehicles

Cleaning and disinfection of transport vehicles in the water of nearby rivers was common in both countries (Vietnam: 67.4%, 29/43; Indonesia: 49.1%, 27/55; *p* = 0.10). However, the most common location for cleaning transport vehicles in Indonesia was inside houses or garages (67.3%, 37/55), but this was less common in Vietnam (25.6%, 11/43; *p* < 0.001). Car washes were more utilized by Indonesian transporters compared to Vietnamese transporters (27.3%, 15/55 vs. 9.3%, 4/43; *p* = 0.038).

**Figure 3:**
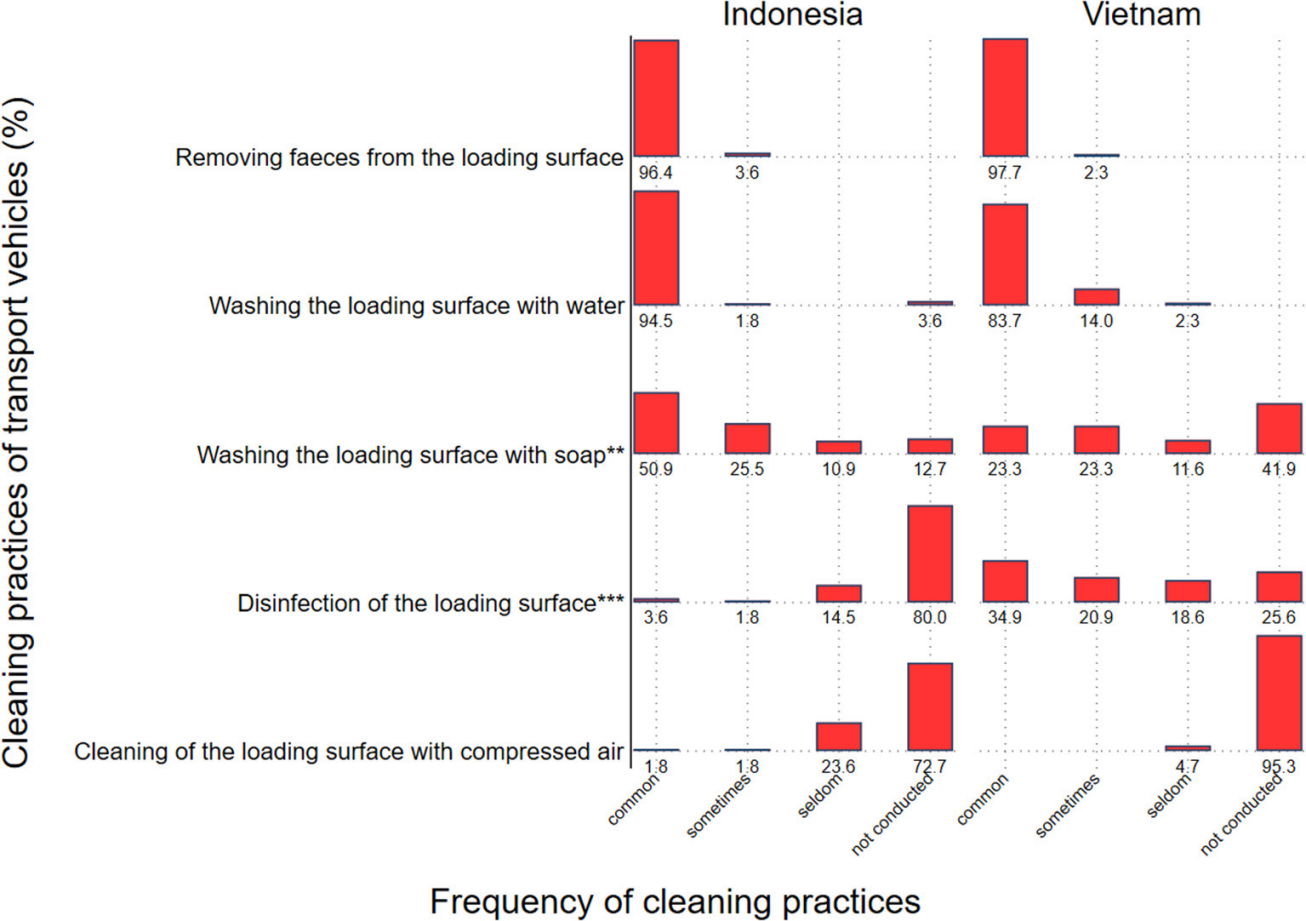
Cleaning practices conducted on transport vehicles in Indonesia (*N* = 55) and Vietnam (*N* = 43) with percent of respondents by frequency category. *P*-values for comparisons between countries for each category are presented as follows: ** for *P* ≤ 0.01, *** for *P* ≤ 0.001.

The most common cleaning practices, i.e., removing feces from loading surfaces and washing loading surfaces with water were similar for both countries (*p* = 1; and *p* = 0.10, respectively) (Figure 3). However, the use of soap to wash loading surfaces was more common in Indonesia compared to Vietnam (*p* = 0.0067), while transporters from Vietnam more “commonly” used disinfectant on loading surfaces compared to Indonesia (*p* < 0.001).

### Health of Transporters

Information was also obtained about whether transporters had experienced health problems while loading and transporting ducks. Almost all of transporter respondents from Indonesia (98.2%; *N* = 54) and Vietnam (95.3%; *N* = 41) indicated that they did not experience any health problems (*p* = 0.58).

## Discussion

This study investigated factors involved in the transportation of moving duck flocks in Vietnam and Indonesia that might facilitate the transmission of avian pathogens such as HPAI virus, and compared the magnitude of these factors between the two countries. While many practices were common to both countries, there were significant differences between the occurrence of some factors, which might help to identify strategies to reduce HPAI transmission during the transport of ducks in each country.

Compared to Vietnamese transporters, Indonesian transporters had more varied sources of income that contributed to a substantial portion of their financial needs and they were more likely to travel to many different locations, such as markets and hatcheries. The more frequent use of transport vehicles for different purposes may have implications for direct (if ducks are transported at the same time with ducks from other farms or other animal species) or indirect (if cleaning is not conducted properly between transports session) spread of HPAI virus (16, 17, 20). Also, considerably more transporters from Indonesia engaged in buying/selling of ducks in markets as an additional income source. This is a concern because wet markets facilitate interspecies transmission of HPAI virus and are considered to be the most likely source for HPAI outbreaks (16, 18, 21, 22). In contrast, transporters in Vietnam mainly specialized in transporting ducks between scavenging locations and did not generally use transport vehicles for other purposes. Although distances traveled per journey with trucks were similar between both countries, the total distance traveled per year was six times larger in Vietnam and more duck flocks and more ducks per load were transported in Vietnam. Compared to Indonesian transporters, Vietnamese transporters more frequently transported other duck flocks on their return journeys (although not significant at *p* < 0.05), thus proving potential opportunities for dissemination of avian pathogens if surface areas of transport vehicles were not properly cleaned and disinfected (16, 17, 23). Adding to this is the fact that transport vehicles were more commonly stored and left open during transport in Vietnam which suggests a higher likelihood for transport vehicles and duck flocks to be exposed to wild birds which could be harboring or excreting avian pathogens such as HPAI virus (15, 23, 24).

In general, collection and delivery of ducks to markets was not very common for transporters in either Indonesia or Vietnam. This indicates that the collection of ducks and delivery to markets is not in their domain, and is more likely conducted by middle men or traders, who have their own vehicles and collect moving ducks from scavenging areas or from farms. Similar observations were made by Meyer et al. (10) and Meyer et al. (20), who surveyed duck farmers in Vietnam.

Differing from the findings of Henning et al. (9) and Meyer et al. (10), our study found that an overwhelming majority of transporters from Indonesia and Vietnam clean their vehicles regularly after each journey, although both previous studies focussed on duck farmers and not directly on transporters of moving duck flocks. Almost all transporter respondents from both Indonesia and Vietnam removed feces and washed the vehicle loading surfaces, with transporters in Vietnam also commonly using disinfectant. This indicates that transporters recognize to a certain extent, the importance of biosecurity practices to prevent the spread of avian pathogens such as HPAI virus. However, further education to increase the biosecurity awareness among actors within the poultry industry is needed, in particular in HPAI endemically infected countries (25–28).

Transporters from Indonesia more frequently experienced deaths of ducks due to disease during the journeys, while transporters from Vietnam more frequently experience deaths of ducks due to dehydration. This difference may be explained by the fact that Indonesian transporters had more opportunities of direct and indirect contact between ducks as birds were sourced and delivered to a wider range of locations; while transporters from Vietnam generally traveled longer distances resulting in potentially longer stressful periods for ducks.

With regards to the disposal of duck carcasses, guidelines from international organizations recommend burial, composting, incineration, rendering or landfill disposal as they are effective in mitigating virus spread and minimizes public health and environmental effects (25, 29, 30). However, despite education campaigns conducted in Indonesia (25), the preferred method of carcass disposal by Indonesian transporters, was to throw carcasses into the environment (in particular into rivers), which has been described previously (31). This increases the likelihood of direct contact of other birds with the carcasses (31) or that scavengers such as roaming dogs open up carcasses and potentially increase virus exposure in the environment (when carcasses are infectious).

Additionally, it also presents a public health risk if untreated river water is consumed by people, especially given that around 4% of households in Indonesia rely on rivers as their main water supply (32–34). Interestingly, a substantial percentage of transporters from Vietnam indicated appropriate methods of disposal including incineration and burial. However, a sizeable proportion of respondents indicated that they prefer to give away the carcasses that died during transport to neighbors (the carcasses may eventually be consumed by the neighbors) or keep them for consumption within their own household. Corroborating with Manabe et al. (28), this suggests that despite a reasonably high awareness of H5N1 infection, Vietnamese transporters adhered to traditional habits. This may be due to insufficient knowledge about the risks of HPAI virus infection, compounded by financial hardships.

None of the transporters involved in this study was using Personal Protective Equipment (PPE), during handling of ducks or cleaning of transport vehicles. Previous research highlighted that lack of awareness and training, but also that low income influences the under-use of protective equipment in developing countries (35).

### Data Limitations

It is difficult to determine if the cohort of transporters recruited into the study is representative of the spatio-temporal distribution of transporters in both countries. This is due to limited literature describing nationwide spatio-temporal distribution of moving duck flocks in both Indonesia and Vietnam and the non-existence of a sampling frame of transporters for both countries. Government lists of moving duck farmers, let alone transporters of moving duck flocks do not exist in either country. The snowball sampling strategy used in this study was the only methodology that allowed us to overcome this problem. It has been previously noted that duck farming is widespread in West and Central Java in Indonesia; and highly concentrated around the Mekong Delta region in Vietnam (10, 17, 36, 37). Therefore, the use of Central Java and the Mekong Delta regions provided a good representation in terms of concentration of duck farming activities in those countries.

The use of survey questionnaires introduced biasness of various forms: recall bias, as the journeys may have been conducted many months earlier resulting in errors in recording journey characteristics and parameters; and social desirability bias, such as that transporters may be unwilling to disclose past experiences of ill-health after handling ducks; and that they may erroneously reported a greater frequency of disinfecting their transport vehicles than in reality. However, we used well-trained interviewers in this study and we are confident that the information we collected is reliable.

Finally, the data summarized here were collected in 2009. Some might argue that there could be changes to the duck industry since then. However, based on the value chain analysis elucidated by Meyer et al. (20) and further described by Kasim et al. (38), our description of the relationship between duck farmers and transporters appears to be a system that exists till this day.

## Conclusion

In conclusion, while HPAI is endemic in both Indonesia and Vietnam, known risk factors that perpetuate HPAI in duck farming differ in importance between the two countries. This is due to dissimilarities in the duck farming industries between the two countries that impact the mode of transport used, movement patterns, disposal methods, and cleaning and disinfection approaches. While practices associated with higher biosecurity risk in Indonesia are related to the multipurpose usage of transport vehicles and the disposal of birds in the environment, unsafe practices in Vietnam relate to the potential mixing of birds during transport, the processing of dead carcasses and the storage and cleaning of transport vehicles.

## Data Availability Statement

The original data presented in this study are included in the article's Supplementary Material (Data Sheets 4, 5). Further inquiries can be directed to the corresponding author.

## Ethics Statement

Ethical approval for interviews with human participants in this study was not provided because the study design for this research was reviewed and approved in accordance with local legislation and institutional requirements in Indonesia and Vietnam. In Indonesia, this was conducted by the Disease Investigation Centre (DIC) in Wates, Yogjakarta; and in Vietnam by the Regional Animal Health Centre VI, Ho Chi Minh City. Data collection for this study was conducted in accordance with the accepted survey guidelines for surveillance activities of both organizations. Written informed consent for participation was not required for this study in accordance with the national legislation and the institutional requirements.

## Author Contributions

JH, JM, LTV, and DY: design of the research study. JH, LTV, and DY: data collection. SWSK and JH: data analysis. SWSK, JH, and JM: development of manuscript. All authors contributed to the manuscript and approved the submitted version.

## Conflict of Interest

The authors declare that the research was conducted in the absence of any commercial or financial relationships that could be construed as a potential conflict of interest.

## Supplementary Material

Data Sheet 1 Questionnaire Indonesia.

Data Sheet 2 Questionnaire Vietnam.

Data Sheet 3 Supplementary Figures and Tables.

Data Sheet 4 Raw data Indonesia.

Data Sheet 5 Raw data Vietnam.
