# Supplementary material for: Transport of Moving Duck Flocks in Indonesia and Vietnam: Management Practices That Potentially Impact Avian Pathogen Dissemination
Source: Front Vet Sci. 2021 Jul 9;8:673624. doi: 10.3389/fvets.2021.673624 (PMC8299275; doi:10.3389/fvets.2021.673624)
Supplement: Data Sheet 2 — Questionnaire Vietnam. [file Data_Sheet_2.pdf]

Australian Government
Australian Centre for International Agricultural Research

THE UNIVERSITY OF QUEENSLAND

TRANSPORT PROVIDER QUESTIONNAIRE VIETNAM

Date of interview:

Name and position of enumerator:

Moving duck flock owners who specified the provider (completed by RAHO VI)

Name of moving duck flock owners	**1.**
	**2.**
	**3.**
Farm reference number of moving duck owners	**1.**
	**2.**
	**3.**
Home (original) location of moving duck owners: *province/district/sub-district/commune/village*	**1.**
	**2.**
	**3.**

Details on the transport provider (from here onwards completed by interviewer)

Name of transport provider	
Age of transport provider	
Home (original) location of transport provider: *province/district/sub-district/commune/village*	
GPS reading of home (original) location of transport provider	

General information on the transport provider

1. What is your main source of income? *Tick one box per row.*

	Very important	Important	Not very important	Not applicable
Transport of ducks to scavenging locations	□	□	□	□
Transport of ducks to markets	□	□	□	□
Transport of ducklings to or from hatcheries	□	□	□	□
Transport of chickens to markets	□	□	□	□
Transport of other animals to markets. *Specify animals* ………………………………	□	□	□	□
Transport of feed. *Specify types of feed* ………………………………	□	□	□	□
Transport of other items. *Specify items* ………………………………	□	□	□	□
Other sources of income *Specify types*<br>1………………………………<br>2………………………………<br>3………………………………	□<br>□<br>□	□<br>□<br>□	□<br>□<br>□	□<br>□<br>□

Comments by the provider of transport to any of these questions:

2. Do you own farming land? If yes, what is the total size of your farming area? *Please specify the unit of measurement (e.g. hectares, square metres etc.) and what you grow.*

3. Do you own ducks? *If yes, please specify if you keeping stationary ducks or moving ducks (enumerator to explain the difference) and provide details on each flock.*

Type	Flock Nr.	Number of ducks in each flock	Age of ducks in each flock (in days)	Breed of ducks in each flock	Specify purpose of each flock (e.g. layer)	HPAI experience in the past – indicate month and year
Stationary ducks	1					
	2					
	3					
Moving ducks	1					
	2					
	3					

4. Do you grow rice? ◻ Yes ◻ No

If yes, please list the periods when you grow rice (months from planting to harvest) and indicate the months when you use these paddies for scavenging by YOUR OWN DUCKS or by OTHER DUCKS (e.g. ducks you transport). Tick the box if used by group of ducks.

Rice cycle	*planting*	*harvest*	*Used for scavenging*	*by own ducks*	*other ducks*
Cycle 1: from	_______to_______		from ______to_______	◻	◻
Cycle 2: from	_______to_______		from ______to_______	◻	◻
Cycle 3: from	_______to_______		from ______to_______	◻	◻

Details on transport provided for ducks

5. **Over the last 12 months** what type of transport have you provided for ducks? *Give details. If you use more than one vehicle per type provide details for each vehicle.*

Transport types	*Tick box if yes*	Number of vehicles	Owned/ rented (*Provide details)*	Floor size in square metres	Floor levels (*Provide details)*	Open/ closed (*Provide details)*	Max N of ducks you are able to transport	Age of ducks transport -ted with this type	Picture taken (tick box if yes) and specify picture number
Trucks	◻								◻ ……...
Boats	◻								◻ ……...
Cars	◻								◻ ……...
Motorbikes	◻								◻ ……..
Bicycles	◻								◻
Others *Specify* …………..	◻								◻ …….

Comments by the provider of transport to any of these questions:

ALL FOLLOWING QUESTIONS REFER TO SCAVENING DUCKS ONLY

6. For how long have you been providing transport for scavenging duck flocks? Specify for each type of transport when you started using this type of transport and if you still using it (or when you stopped using it). *Indicate why you prefer or stopped using each type.*

Transport types	Used from	Used until	Reasons why you prefer this type of transport (or why you stopped providing this type of transport)

7. Please indicate the **number of scavenging duck flocks** you are (or were) transporting **per year** with each type of transport you providing.

Transport types	Minimum number of duck flocks per year	Average number of duck flocks per year	Maximum number of duck flocks per year

8. Please provide details on the **number of scavenging duck farms** you are (or were) visiting **to obtain one transport load** for each type of transport**.**

Transport types	Minimum number of duck farms visited	Average number of duck farms visited	Maximum number of duck farms visited

9. Provide details on the **combination of and the possible contact (direct physical contact OR indirect contact by faeces, excretions or secretions) of scavenging duck flocks** for each type of transport. *Tick the appropriate boxes.*

Transport types	Transport of duck flocks from different farmers in a single load	Direct or indirect contact between different duck flocks	Transport of ducks of different age groups in a single load
	□	□	□
	□	□	□
	□	□	□

10. Provide details on the scavenging duck flocks you have transported in the last 12 months.

Transport types	Details	Number of duck flocks transported per load			Number of ducks transported per load			Age of ducks transported (in days)			Payment (in VND) received (specify if per load/flock/bird)		
		Min	Ave	Max	Min	Ave	Max	Min	Ave	Max	Min	Ave	Max
	Numbers										per…….	per…….	per…….
	Months												
	Numbers										per…….	per…….	per…….
	Months												
	Numbers										per…….	per…….	per…….
	Months												

Comments by the provider of transport to any of these questions:

11. If you have purchased a transport vehicle, specify how much you paid for what type of transport vehicle in what year?

Transport types	Purchase price (in VND)	Purchased when (specify year)

12. If you rent or lease a transport vehicle specify how much you pay for it? Specify the period of payment (per month, per year, etc.)?

Transport types	Rental price (in VND)	Rented since when (specify year)
	per………..	
	per………..	
	per………..	

13. Specify the items (e.g. petrol, oil, disinfectant, wages to drivers, wages to workers etc.) you have to purchase or provide for when transporting ducks? List the **average cost per year** for each item specified.

Transport types	Item per transport type	Average cost per item per year (in VND)
	1.	
	2.	
	3.	
	4.	
	1.	
	2.	
	3.	
	4.	
	1.	
	2.	
	3.	
	4.	

14. Provide details on the journeys when you transported ducks to scavenging locations.

Transport types	Details	Number of journeys per year			Distance travelled per year (in km)			Distance travelled per journey (in km)			Duration per journey (in hours)		
		Min	Ave	Max	Min	Ave	Max	Min	Ave	Max	Min	Ave	Max
	Numbers												
	Months												
	Numbers												
	Months												
	Numbers												
	Months												

15. Indicate the type of journeys you conduct with scavenging ducks? *Tick one box per row.*

	Very often	Sometimes	Seldom	Not applicable
Take ducks to scavenging locations and return empty	□	□	□	□
Take ducks to scavenging locations and return with other ducks	□	□	□	□
Take ducks to scavenging locations and return with other load. *Specify load……………………………*	□	□	□	□
Other types of journeys. *Specify these types of journeys* . ………………………………	□	□	□	□

Comments by the provider of transport to any of these questions:

16. Provide details from where do you collect duck flocks (source). *Tick one box per row.*

Source of collection	Common	Sometimes	Not common	Never	Months of most frequent use
Rice field paddies	□	□	□	□	
Duck farms	□	□	□	□	
Markets	□	□	□	□	
Village area	□	□	□	□	
Hatcheries	□	□	□	□	
Other. *Specify.* ………………………..	□	□	□	□	

17. Provide details to where you transport duck flocks (destination). *Tick one box per row.*

Destination of release	Common	Sometimes	Not common	Never	Months of most frequent use
Rice field paddies	□	□	□	□	
Duck farms	□	□	□	□	
Markets	□	□	□	□	
Village area	□	□	□	□	
Hatcheries	□	□	□	□	
Other. *Specify.* ………………………..	□	□	□	□	

18. Provide details on items you transport **together with ducks**. *Tick one box per row.*

	Common	Sometimes	Not common	Never	Months of most frequent use
Chickens	□	□	□	□	
Other birds *Specify*………………	□	□	□	□	
Other animals *Specify*………………	□	□	□	□	
Duck feed *Specify*………………	□	□	□	□	
Other animal feed *Specify*………………	□	□	□	□	
Eggs	□	□	□	□	
Other items *Specify*………………	□	□	□	□	

Care provided for ducks during and after transport

19. How do you care for the scavenging ducks during transport?

Care during transport	Tick if provided	List details
Provide water	□	
Provide feed	□	
Spray birds with water	□	
Resting stops	□	
Other S*pecify* …………………………	□	

20. Specify what procedures are conducted at the arrival of ducks in the scavenging areas?

Health and mortalities of ducks transported

21. What symptoms have you observed in scavenging ducks that became sick while they were transported? *Please describe.*

Comments by the provider of transport to any of these questions:

22. What are the causes of death in scavenging ducks while they are transported? *Tick one box per row. List main types for each cause.*

	Common	Sometimes	Not common	Never	Provide details (type of disease, injury etc.)
Disease	□	□	□	□	
Dehydration	□	□	□	□	
Injury during transport	□	□	□	□	
Other. S*pecify*………..	□	□	□	□	
Unknown	□	□	□	□	

23. How many deaths per load of scavenging ducks transported have you had in the past? *Provide details for each transport type.*

Transport types	Minimum number of duck deaths per load	Average number of duck deaths per load	Maximum number of duck deaths per load

24. What is done with carcasses of scavenging ducks that died during the transport? *Tick the appropriate boxes.*

Burned	□	Buried	□
Sold	□	Household consumption	□
Processed for fish feed	□	Thrown away	□
Given to neighbours	□	Other. S*pecify*………………………	□

25. Do you get financially punished by the duck flock owner if scavenging ducks die during the transport? *Provide details.*

Cleaning, disinfection and storage of the transport vehicle

26. Which of the following cleaning practices do you conduct on your transport vehicle?

Cleaning practice	Common	Sometimes	Seldom	Never	Details (per transport type)
Removing of faeces from the loading surface	□	□	□	□	What is done with the faeces?
Washing the loading surface with water	□	□	□	□	What is done with the water?
Use of soap for washing the loading surface	□	□	□	□	What disinfectant is used?
Disinfection of the loading surface	□	□	□	□	What disinfectant is used?
Use of gas on the loading surface	□	□	□	□	What gas is used?
Other *Specify* ………………………	□	□	□	□	

27. Do you conduct additional procedures when you had many deaths of scavenging ducks during transport? *Provide details.*

28. Where do you conduct the cleaning or disinfection of the transport vehicle?

**Location of cleaning**	***Tick if used***	**Provide details (per transport type)**
In a house/garage	□	
In a river	□	
In a village	□	
In a car wash	□	
Other S*pecify*……………………..	□	

Comments by the provider of transport to any of these questions:

29. Where do you store the transport vehicle?

Location	*Tick if used*	Provide details (per transport type)	Vehicle exposed to wild birds *(tick if yes). Specify type of wild birds.*
House/garage	□		□ ………………………
Outside	□		□ ………………………
Other *Specify*…………	□		□………………………

People involved, permits required and other information on transport

30. Provide details on the number of people involved in the transport of scavenging ducks and the payment they receive.

Activities conduced by people involved in the transport of ducks	Number of people *(provide number)*	Payment received in VND *(indicate if per bird, per flock, per hour, per day)*	Payment conducted by transport provider or by the duck owner (*specify)*
Collection of ducks			
Loading of ducks			
Driving of the vehicle			
Release of ducks at the scavenging location			
Other people involved *Specify for what activity* …………….……………..			

31. Who is travelling together with driver in the transport vehicle to the duck scavenging location? *Please tick one box per row.*

	Common	Sometimes	Seldom	Never
Duck herder/worker	□	□	□	□
Duck owner	□	□	□	□
Other. *Specify*…………..	□	□	□	□

32. Do you require permits to transport scavenging ducks? □ Yes □ No
If yes, provide the following details.
A) From where do you obtain the permits?
B) For how long are the permits valid?
C) What does the permit specify? The area where you are working? The time period where you are working? Or what else?
D) Are there any conditions connected to the permits?

34. Do you see changes to the transport of scavenging ducks during the rice harvest season? *Please describe.*

35. What are the main constraints you face when proving transport for scavenging ducks?

Comments by the provider of transport to any of these questions:

36. Have you or any other people involved in the loading and transporting of poultry experienced any health problems (e.g. sore throat after handling of birds, coughing after cleaning of vehicles)? *Provide details.*

Information on purchases and sales of ducks by the transport provider

37. Do you purchase ducks and sell them to markets or to other traders?

□ Yes □ No

If yes, provide the following details.

Where do you keep the birds after you purchased them from the duck farmer?

To whom to you sell your birds? *Please tick one box per row.*

	Very often	Sometimes	Seldom	Never
Sale directly to markets	□	□	□	□
Sale to other traders	□	□	□	□
Sale to other duck owners	□	□	□	□
Other sales. *Specify to whom* . ………………………………	□	□	□	□

Provide details on how many times per year did purchase or sell ducks for what price

Details	Number of times per year when duck flocks are purchased/sold			How much to you pay per duck (in VND)?			For how much do sell a duck (in VND)?		
	Min	Ave	Max	Min	Ave	Max	Min	Ave	Max
Numbers									
Months									

38. Other comments by the provider of the transport:

39. Comments and observations by enumerator:

Comments by the provider of transport to any of these questions:

Signature of interviewee

Thank you for your participation.

Comments by the provider of transport to any of these questions:
