# Supplementary material for: Transport of Moving Duck Flocks in Indonesia and Vietnam: Management Practices That Potentially Impact Avian Pathogen Dissemination
Source: Front Vet Sci. 2021 Jul 9;8:673624. doi: 10.3389/fvets.2021.673624 (PMC8299275; doi:10.3389/fvets.2021.673624)
Supplement: Data Sheet 3 — Supplementary Figures and Tables. [file Data_Sheet_3.pdf]

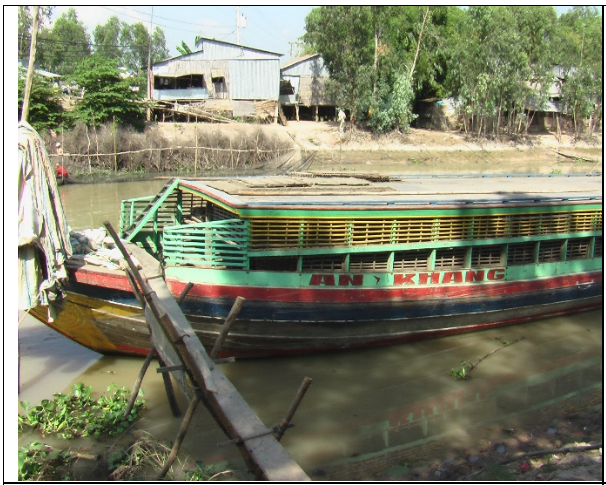

Boat for transporting ducks (Vietnam)

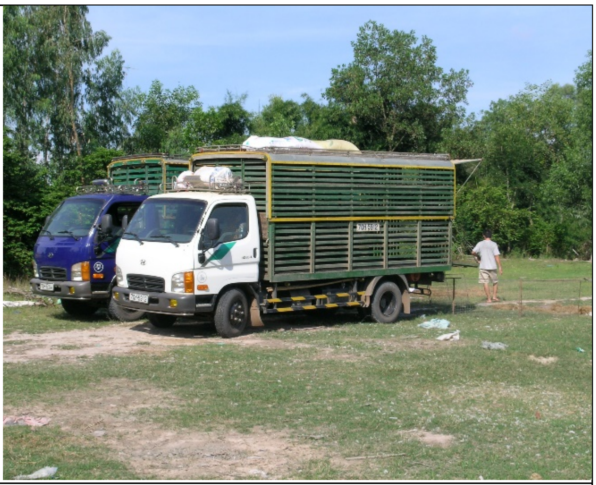

Truck for transporting ducks (Vietnam)

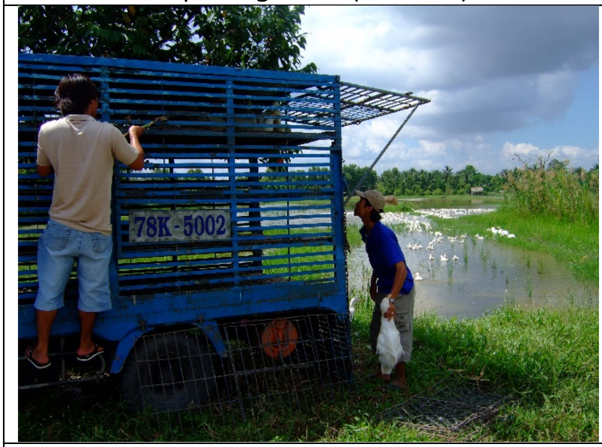

Unloading ducks (Vietnam)

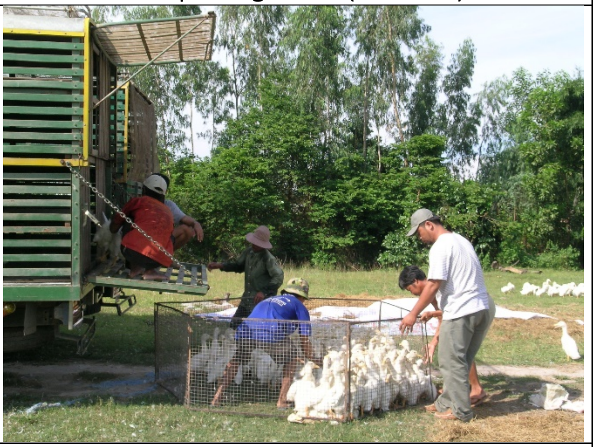

Unloading ducks (Vietnam)

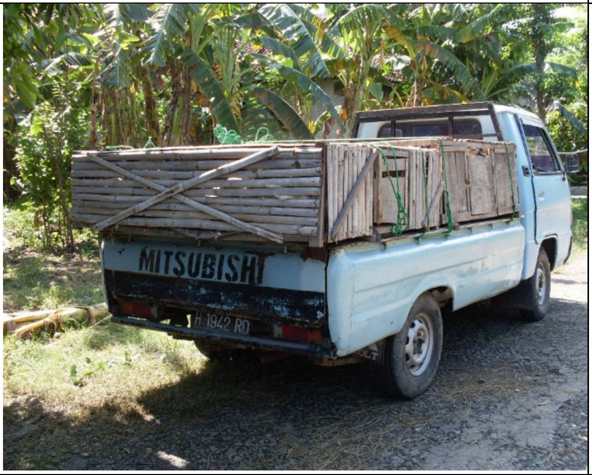

Truck for transporting ducks (Indonesia)

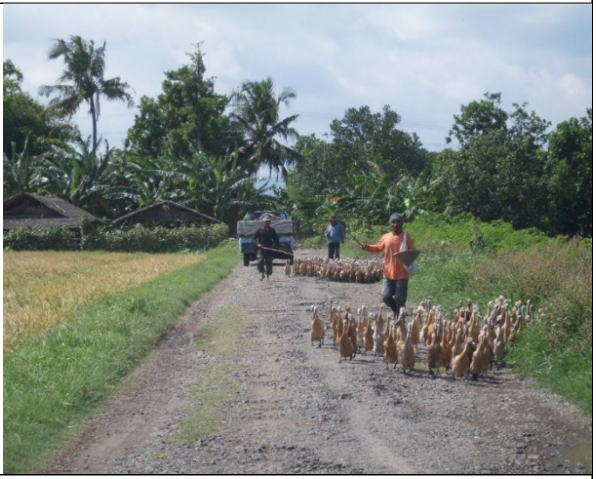

Herding of ducks (Indonesia)

SUPPLEMENTARY FIGURE 1 | Images of types of transport used for moving duck flocks in Vietnam and Indonesia.

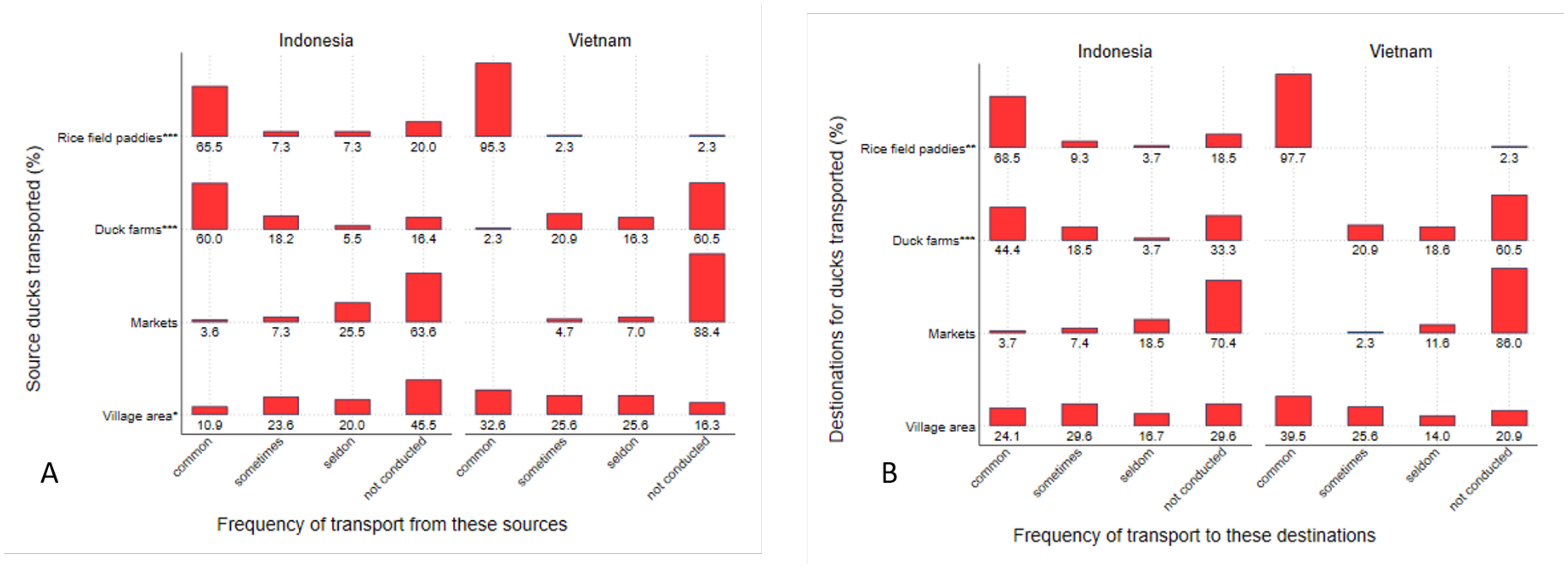

SUPPLEMENTARY FIGURE 2 | (A) Locations from where moving duck flocks were collected by transporters in Indonesia (*N* = 55) and Vietnam (*N* = 43). **(B)** Destinations to where moving duck flocks were delivered by transporters in Indonesia (*N* = 55) and Vietnam (*N* = 43). P-values for comparisons between countries for each category are presented as follows: * for P ≤ 0.05, ** for P ≤ 0.01, *** for P ≤ 0.001.

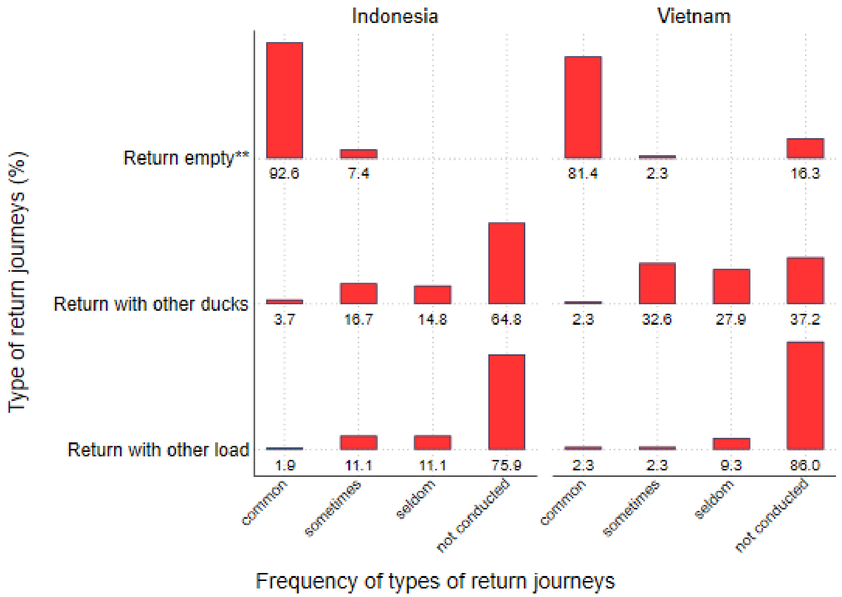

SUPPLEMENTARY FIGURE 3 | Characteristics of return journeys conducted by transporters in Indonesia (N=55) and Vietnam (N=43). P-values for comparisons between countries for each category are presented as follows: ** for P ≤ 0.01.

SUPPLEMENTARY TABLE 1 | Original responses by transport providers (recorded on a 5-scale Likert scale) on importance of transport activities, frequency of transporting items with duck flocks, frequency of deaths of ducks, and frequency of cleaning practices of transporter vehicles in Indonesia (N=55) and Vietnam (N=43).

		**Indonesia % (N)**					**Vietnam % (N)**					
**Importance of transport activities**	**transport activities**	**very important**	**important**	**not important**	**not conducted**	**total**	**very important**	**important**	**not important**	**not conducted**	**total**	**p-value**
	transport of ducks to scavenging locations	60.0 (33)	38.2 (21)	1.8 (1)	0.0 (0)	100.0 (55)	67.4 (29)	27.9 (12)	2.3 (1)	2.3 (1)	100.0 (43)	0.58
	transport of ducks to markets	5.5 (3)	10.9 (6)	27.3 (15)	56.4 (31)	100.0 (55)	2.3 (1)	7.0 (3)	11.6 (5)	79.1 (34)	100.0 (43)	0.38
	transport of ducks to or from hatcheries	5.5 (3)	14.5 (8)	21.8 (12)	58.2 (32)	100.0 (55)	7.0 (3)	0.0 (0)	11.6 (5)	81.4 (35)	100.0 (43)	0.085
	transport of chickens or other animals to markets	0.9 (1)	0.0 (0)	12.7 (14)	86.4 (95)	100.0 (110)	0.0 (0)	1.2 (1)	4.7 (4)	94.2 (81)	100.0 (86)	1
	transport of feed	3.6 (2)	21.8 (12)	16.4 (9)	58.2 (32)	100.0 (55)	0.0 (0)	2.3 (1)	25.6 (11)	72.1 (31)	100.0 (43)	0.0014
	transport of other items	32.7 (18)	16.4 (9)	18.2 (10)	32.7 (18)	100.0 (55)	4.8 (2)	4.8 (2)	11.9 (5)	78.6 (33)	100.0 (42)	<0.001
	other sources of income	49.1 (27)	9.1 (5)	7.3 (4)	34.5 (19)	100.0 (55)	7.0 (3)	16.3 (7)	2.3 (1)	74.4 (32)	100.0 (43)	0.00089
**Frequency of transporting items with duck flocks**	**items transported together with duck flocks**	**common**	**sometimes**	**seldom**	**not conducted**	**total**	**common**	**sometimes**	**seldom**	**not conducted**	**total**	**p-value**
	chickens	5.5 (3)	0.0 (0)	9.1 (5)	85.5 (47)	100.0 (55)	2.3 (1)	0.0 (0)	4.7 (2)	93 (40)	100.0 (43)	0.63
	other animals	0.0 (0)	0.0 (0)	10.9 (6)	89.1 (49)	100.0 (55)	0 (0)	9.3 (4)	4.7 (2)	86 (37)	100.0 (43)	0.034
	duck feed	20 (11)	25.5 (14)	25.5 (14)	29.1 (16)	100.0 (55)	4.7 (2)	23.3 (10)	23.3 (10)	48.8 (21)	100.0 (43)	0.094
	other animal feed	1.8 (1)	3.6 (2)	16.4 (9)	78.2 (43)	100.0 (55)	0.0 (0)	4.7 (2)	7 (3)	88.4 (38)	100.0 (43)	1
	eggs	9.1 (5)	30.9 (17)	14.5 (8)	45.5 (25)	100.0 (55)	0.0 (0)	0.0 (0)	4.7 (2)	95.3 (41)	100.0 (43)	<0.001
	other items	5.5 (3)	9.1 (5)	1.8 (1)	83.6 (46)	100.0 (55)	0.0 (0)	4.7 (2)	0.0 (0)	95.3 (41)	100.0 (43)	0.18

frequency of duck deaths	causes of duck deaths during transport	common	sometimes	seldom	not conducted	total	common	sometimes	seldom	not conducted	total	p-value
	disease	1.8 (1)	14.5 (8)	25.5 (14)	58.2 (32)	100.0 (55)	0.0 (0)	4.7 (2)	4.7 (2)	90.7 (39)	100.0 (43)	0.11
	dehydration	12.7 (7)	18.2 (10)	25.5 (14)	43.6 (24)	100.0 (55)	16.3 (7)	55.8 (24)	18.6 (8)	9.3 (4)	100.0 (43)	<0.001
	injury during transport	7.3 (4)	49.1 (27)	23.6 (13)	20.0 (11)	100.0 (55)	9.3 (4)	44.2 (19)	32.6 (14)	14.0 (6)	100.0 (43)	0.84
	other causes	1.8 (1)	1.8 (1)	25.5 (14)	70.9 (39)	100.0 (55)	0.0 (0)	0.0 (0)	0.0 (0)	100.0 (43)	100.0 (43)	0.5
	unknown	0.0 (0)	0.0 (0)	5.5 (3)	94.5 (52)	100.0 (55)	0.0 (0)	0.0 (0)	0.0 (0)	100.0 (43)	100.0 (43)	1

frequency of cleaning practices	cleaning practices of transport vehicles	common	sometimes	seldom	not conducted	total	common	sometimes	seldom	not conducted	total	p-value
	removing faeces from the loading surface	96.4 (53)	3.6 (2)	0.0 (0)	0.0 (0)	100.0 (55)	97.7 (42)	2.3 (1)	0.0 (0)	0.0 (0)	100.0 (43)	1
	washing the loading surface with water	94.5 (52)	1.8 (1)	0.0 (0)	3.6 (2)	100.0 (55)	83.7 (36)	14 (6)	2.3 (1)	0.0 (0)	100.0 (43)	1
	washing the loading surface with soap	50.9 (28)	25.5 (14)	10.9 (6)	12.7 (7)	100.0 (55)	23.3 (10)	23.3 (10)	11.6 (5)	41.9 (18)	100.0 (43)	0.0031
	disinfection of the loading surface	3.6 (2)	1.8 (1)	14.5 (8)	80 (44)	100.0 (55)	34.9 (15)	20.9 (9)	18.6 (8)	25.6 (11)	100.0 (43)	<0.001
	cleaning of the loading surface with compressed air	1.8 (1)	1.8 (1)	23.6 (13)	72.7 (40)	100.0 (55)	0.0 (0)	0.0 (0)	4.7 (2)	95.3 (41)	100.0 (43)	0.5

frequency of transport from these sources	source ducks transported	common	sometimes	seldom	not conducted	total	common	sometimes	seldom	not conducted	total	p-value
	rice field paddies	65.5 (36)	7.3 (4)	7.3 (4)	20 (11)	100.0 (55)	95.3 (41)	2.3 (1)	0.0 (0)	2.3 (1)	100.0 (43)	<0.001
	duck farms	60.0 (33)	18.2 (10)	5.5 (3)	16.4 (9)	100.0 (55)	2.3 (1)	20.9 (9)	16.3 (7)	60.5 (26)	100.0 (43)	<0.001
	markets	3.6 (2)	7.3 (4)	25.5 (14)	63.6 (35)	100.0 (55)	0.0 (0)	4.7 (2)	7.0 (3)	88.4 (38)	100.0 (43)	0.46
	Village areas#	10.9 (6)	23.6 (13)	20.0 (11)	45.5 (25)	100.0 (55)	32.6 (14)	25.6 (11)	25.6 (11)	16.3 (7)	100.0 (43)	0.025

frequency of transport to these destinations	destinations for ducks transported	common	sometimes	seldom	not conducted	total	common	sometimes	seldom	not conducted	total	p-value
	rice field paddies	68.5 (37)	9.3 (5)	3.7 (2)	18.5 (10)	100.0 (54)	97.7 (42)	0.0 (0)	0.0 (0)	2.3 (1)	100.0 (43)	0.0054
	duck farms	44.4 (24)	18.5 (10)	3.7 (2)	33.3 (18)	100.0 (54)	0.0 (0)	20.9 (9)	18.6 (8)	60.5 (26)	100.0 (43)	<0.001

	markets	3.7 (2)	7.4 (4)	18.5 (10)	70.4 (38)	100.0 (54)	0.0 (0)	2.3 (1)	11.6 (5)	86.0 (37)	100.0 (43)	0.13
	Village areas#	24.1 (13)	29.6 (16)	16.7 (9)	29.6 (16)	100.0 (54)	39.5 (17)	25.6 (11)	14.0 (6)	20.9 (9)	100.0 (43)	0.3

**frequencies of types of return journeys**	**type of return journeys**	**common**	**sometimes**	**seldom**	**not conducted**	**total**	**common**	**sometimes**	**seldom**	**not conducted**	**total**	**p-value**
	return empty	92.6 (50)	7.4 (4)	0.0 (0)	0.0 (0)	100.0 (54)	81.4 (35)	2.3 (1)	0.0 (0)	16.3 (7)	100.0 (43)	0.0025
	return with other ducks	3.7 (2)	16.7 (9)	14.8 (8)	64.8 (35)	100.0 (54)	2.3 (1)	32.6 (14)	27.9 (12)	37.2 (16)	100.0 (43)	0.17
	return with other load	1.9 (1)	11.1 (6)	11.1 (6)	75.9 (41)	100.0 (54)	2.3 (1)	2.3 (1)	9.3 (4)	86.0 (37)	100.0 (43)	0.29

# represents areas other than duck farms that contain ducks within village boundaries
